# Supplementary material for: Epistatic interactions between oxytocin- and dopamine-related genes and trust
Source: PLoS One. 2024 Sep 19;19(9):e0308728. doi: 10.1371/journal.pone.0308728 (PMC11412487; doi:10.1371/journal.pone.0308728)
Supplement: S1 Table — (DOCX) [file pone.0308728.s001.docx]

Epistatic interactions between oxytocin- and dopamine-related genes and trust

Supplementary materials

Table of contents

S1 Table. Frequency and distribution of genotypes of oxytocin- and dopamine-related genes

S2 Table. Trust questionnaire items

S3 Table. Epistatic interaction effects between oxytocin- and dopamine-related genotypes on trust

S4 Table. Trust mean score by genotyped groups

S1 Table. Frequency and distribution of genotypes of oxytocin- and dopamine-related genes

| SNP | Minor allele | Major allele | Minor allele frequency | Hardy-Weinberg equilibrium p-value ^a^ |
| --- | --- | --- | --- | --- |
| *OXTR* rs53576 | G | A | 0.36 | 0.14; 0.45 |
| *OXTR* rs2254298 | A | G | 0.28 | 0.12; 1.00 |
| *OXTR* rs1042778 | T | G | 0.10 | 1.00; 1.00 |
| *CD38* rs3796863 | A | C | 0.37 | 0.57; 0.16 |
| *COMT* rs4680 | A | G | 0.36 | 0.88; 0.70 |
| *DRD2* rs1800497 | T | C | 0.35 | 0.19; 0.69 |

^a^ P-values are presented for women and men, separately.
