# Supplementary material for: Epistatic interactions between oxytocin- and dopamine-related genes and trust
Source: PLoS One. 2024 Sep 19;19(9):e0308728. doi: 10.1371/journal.pone.0308728 (PMC11412487; doi:10.1371/journal.pone.0308728)
Supplement: S2 Table — (DOCX) [file pone.0308728.s002.docx]

S2 Table. Trust questionnaire items

|  | Items | Mean (SD) |
| --- | --- | --- |
| 1 | Do you think most people are trying to help others, or do you think they are only thinking about themselves? | 0.59 (0.22) |
| 2 | Do you think most people are trustworthy? | 0.69 (0.26) |
| 3 | Do you think people in your neighborhood trust each other? | 0.56 (0.28) |
| 4 | Do you think your neighbors are helping each other? | 0.55 (0.27) |
| 5 | How much do you trust your neighbors? | 0.62 (0.24) |
| 6 | Do you find it annoying to get to know your neighbors? a | 0.61 (0.26) |
| 7 | How much do you trust government agencies? | 0.68 (0.24) |
| 8 | How much do you trust the police? | 0.77 (0.19) |
| 9 | How much do you trust medical institutions? | 0.62 (0.23) |
| 10 | How much do you trust newspapers and TV? | 0.58 (0.24) |

The score of each item ranged from 0 to 1.

^a^ The response to this question was reversed.

Item 1-2 represent general trust, item 3-6 represent neighbourhood trust, and item 7-10 represent institutional trust.
