# Supplementary material for: Epistatic interactions between oxytocin- and dopamine-related genes and trust
Source: PLoS One. 2024 Sep 19;19(9):e0308728. doi: 10.1371/journal.pone.0308728 (PMC11412487; doi:10.1371/journal.pone.0308728)
Supplement: S3 Table — (DOCX) [file pone.0308728.s003.docx]

S3 Table. Epistatic interaction effects between oxytocin- and dopamine-related genotypes on trust

|  | General trust | |  | Neighborhood trust | |  | Institutional trust | |
| --- | --- | --- | --- | --- | --- | --- | --- | --- |
|  | B | P-value |  | B | P-value |  | B | P-value |
| *OXTR* rs53576 × *COMT* rs4680 | 10.64 | 0.08 |  | 4.71 | 0.42 |  | 5.62 | 0.21 |
| *OXTR* rs53576 × *COMT* rs4680 × sex | -18.72 | 0.10 |  | -0.92 | 0.93 |  | 3.45 | 0.69 |
|  |  |  |  |  |  |  |  |  |
| *OXTR* rs2254298 × *COMT* rs4680 | -2.79 | 0.64 |  | 1.87 | 0.75 |  | 5.88 | 0.23 |
| *OXTR* rs2254298 × *COMT* rs4680 × sex | 16.95 | 0.10 |  | 5.86 | 0.54 |  | -6.83 | 0.48 |
|  |  |  |  |  |  |  |  |  |
| *OXTR* rs1042778 × *COMT* rs4680 | 7.81 | 0.27 |  | 1.16 | 0.88 |  | 4.25 | 0.47 |
| *OXTR* rs1042778 × *COMT* rs4680 × sex | -1.36 | 0.89 |  | **26.38** | **0.02** |  | 12.32 | 0.16 |
|  |  |  |  |  |  |  |  |  |
| *CD38* rs3796863 × *COMT* rs4680 | 0.48 | 0.94 |  | 1.81 | 0.76 |  | 4.23 | 0.33 |
| *CD38* rs3796863 × *COMT* rs4680 × sex | -12.19 | 0.24 |  | -2.44 | 0.79 |  | -11.09 | 0.21 |
|  |  |  |  |  |  |  |  |  |
| *OXTR* rs53576 × *DRD2* rs1800497 | 6.55 | 0.23 |  | 1.25 | 0.77 |  | 3.99 | 0.24 |
| *OXTR* rs53576 × *DRD2* rs1800497 × sex | -0.48 | 0.96 |  | 4.45 | 0.51 |  | 0.72 | 0.90 |
|  |  |  |  |  |  |  |  |  |
| *OXTR* rs2254298 × *DRD2* rs1800497 | 5.79 | 0.29 |  | 6.49 | 0.28 |  | -0.06 | 0.99 |
| *OXTR* rs2254298 × *DRD2* rs1800497 × sex | -12.61 | 0.23 |  | -5.14 | 0.58 |  | **-21.04** | **0.01** |
|  |  |  |  |  |  |  |  |  |
| *OXTR* rs1042778 × *DRD2* rs1800497 | 1.70 | 0.81 |  | -13.36 | 0.11 |  | -9.80 | 0.13 |
| *OXTR* rs1042778 × *DRD2* rs1800497 × sex | -4.37 | 0.68 |  | 10.91 | 0.38 |  | 10.38 | 0.27 |
|  |  |  |  |  |  |  |  |  |
| *CD38* rs3796863 × *DRD2* rs1800497 | 5.18 | 0.41 |  | 2.07 | 0.69 |  | -1.29 | 0.78 |
| *CD38* rs3796863 × *DRD2* rs1800497 × sex | -4.89 | 0.62 |  | -5.06 | 0.60 |  | 1.27 | 0.88 |

All models were adjusted for age.

Standard errors were calculated with a sandwich variance estimator.
