# Supplementary material for: Epistatic interactions between oxytocin- and dopamine-related genes and trust
Source: PLoS One. 2024 Sep 19;19(9):e0308728. doi: 10.1371/journal.pone.0308728 (PMC11412487; doi:10.1371/journal.pone.0308728)
Supplement: S4 Table — (DOCX) [file pone.0308728.s004.docx]

S4 Table. Trust mean score by genotyped groups

|  | Mean (SD) | Mean (SD) | Mean (SD) | Mean (SD) |
| --- | --- | --- | --- | --- |
| Neighborhood trust score | | | | |
| *OXTR* rs1042778 + *COMT* rs4680 | GG + AG/AA (n=54) | TG/TT + AG/AA (n=20) | GG + GG (n=34) | TG/TT + GG (n=6) |
|  | 57.8 (21.1) | 49.8 (24.6) | 54.0 (22.7) | 72.6 (14.6) |
|  |  |  |  |  |
| Institutional trust score | | | | |
| *OXTR* rs2254298 + *DRD2* rs1800497 | AG/AA + TT/TC (n=32) | GG + TT/TC (n=40) | AG/AA + CC (n=17) | GG + CC (n=25) |
|  | 54.5 (22.4) | 65.0 (16.2) | 71.0 (16.4) | 59.5 (20.6) |
