# Supplementary material for: Epistatic interactions between oxytocin- and dopamine-related genes and trust
Source: PLoS One. 2024 Sep 19;19(9):e0308728. doi: 10.1371/journal.pone.0308728 (PMC11412487; doi:10.1371/journal.pone.0308728)
Supplement: Attachment — Submitted filename: PLOS (1).pdf [file pone.0308728.s005.pdf]

The manuscript by Koyama et al. attempts to establish a link between the oxytocinergic and dopaminergic systems, hypothesizing an epistatic relationship between the corresponding genes. These gene sets play key roles in shaping behaviours of key societal significance.

The study's findings highlight the presence of an interaction between OXTRrs1042778 and COMTrs4680 concerning neighbourhood trust. An interaction was also observed between OXTRrs2254298 and DRD2rs1800497 impacting institutional trust, but exclusively among males.

While these results, albeit limited, may be of interest to a specialized audience, the manuscript is not without its limitations. Firstly, the authors devote scant attention to the literature on CD38 and its role in confidence, a subject extensively discussed by biologists and medical groups working in different fields. Moreover, references to most of the pivotal papers on the subject are conspicuously absent.

Another limitation is the failure to analyse the results in relation to the sex of the subjects. A second, and arguably more critical limitation, is the exclusive focus on Japanese individuals in the analysis. While this is not problematic *per se* and may contribute valuable insights within the context of Japan, it makes the conclusions difficult to extrapolate to societies lacking the peculiar characteristics of Japanese society. These factors hinder the extension of the study's results to a broader audience.

The authors acknowledge additional limitations at the conclusion of an extensive discussion.
